# Effects of particulate matter on the pulmonary and vascular system: time course in spontaneously hypertensive rats

**DOI:** 10.1186/1743-8977-2-2

**Published:** 2005-03-24

**Authors:** Miriam E Gerlofs-Nijland, A John F Boere, Daan LAC Leseman, Jan AMA Dormans, Thomas Sandström, Raimo O Salonen, Leendert van Bree, Flemming R Cassee

**Affiliations:** 1 National Institute for Public Health and the Environment (RIVM), P.O. Box 1, 3720 BA, Bilthoven, The Netherlands; 2 Department of Respiratory Medicine and Allergy, University Hospital Umeå, Umeå, Sweden; 3 Department of Environmental Health, National Public Health Institute (KTL), Kuopio, Finland

## Abstract

**Background:**

This study was performed within the scope of two multi-center European Commission-funded projects (HEPMEAP and PAMCHAR) concerning source-composition-toxicity relationship for particulate matter (PM) sampled in Europe. The present study aimed to optimize the design for PM *in vivo *toxicity screening studies in terms of dose and time between a single exposure and the determination of the biological responses in a rat model mimicking human disease resulting in susceptibility to ambient PM. Dust in thoracic PM size-range (aerodynamic diameter <10 μm) was sampled nearby a road tunnel (RTD) using a high volume cascade impactor. Spontaneously hypertensive rats were exposed to urban dust collected in Ottawa, Canada (EHC-93 10 mg/kg of body weight; reference PM) or different RTD doses (0.3, 1, 3, 10 mg/kg of body weight) by intratracheal instillation. Necropsy was performed at 4, 24, or 48 hr after exposure.

**Results:**

The neutrophil numbers in bronchoalveolar lavage fluid increased tremendously after exposure to the highest RTD doses or EHC-93. Furthermore, PM exposure slightly affected blood coagulation since there was a small but significant increase in the plasma fibrinogen levels (factor 1.2). Pulmonary inflammation and oxidative stress as well as changes in blood coagulation factors and circulating blood cell populations were observed within the range of 3 to 10 mg PM/kg of body weight without significant pulmonary injury.

**Conclusion:**

The optimal dose for determining the toxicity ranking of ambient derived PM samples in spontaneously hypertensive rats is suggested to be between 3 and 10 mg PM/kg of body weight under the conditions used in the present study. At a lower dose only some inflammatory effects were detected, which will probably be too few to be able to discriminate between PM samples while a completely different response pattern was observed with the highest dose. In addition to the dose, a 24-hr interval from exposure to sacrifice seemed appropriate to assess the relative toxic potency of PM since the majority of the health effects were observed one day after PM exposure compared to the other times examined. The aforementioned considerations provide a good basis for conducting PM toxicity screening studies in spontaneously hypertensive rats.

## Background

The effects of particulate matter (PM) on human health are a major concern since current epidemiology data show health effects at PM concentrations below common ambient air quality standards or health-based guidelines [1]. Furthermore, a clear association has been demonstrated between increased airborne PM concentrations and exacerbation of chronic respiratory and cardiovascular diseases, respiratory symptoms or decreased lung function, as revealed in increased hospital admissions and emergency room visits or even premature mortality [1].

PM has not been linked to numerous adverse health effects by epidemiological research alone. It is supported by PM toxicity studies in both experimental animals and human volunteers. Inhalation exposure studies have shown that short-term exposure to diesel exhaust has an acute inflammatory effect on normal human airways resulting in marked neutrophilia, activation of mast cells and neutrophils, and the production of cytokines and chemokines associated with neutrophil accumulation and activation [2,3]. In addition, repeated exposures of experimental animals [4,5] and humans [6] to direct real-world ambient air in urban areas of São Paulo, Mexico City and Florence suggest that more adverse health effects occur at locations with high traffic densities, possibly due to the higher level of (fine) PM (aerodynamic diameter <2.5 μm). These studies have demonstrated serious adverse health impacts on the airways, including lesions in the upper and lower airway tissues, increased airway reactivity and immunotoxic effects. Many *in vivo *animal exposure studies have shown the toxicity of PM, sampled from ambient air or diesel exhaust on filters, and administered by intratracheal instillation. PM fractions cause not just airway and lung inflammation, and injury [7-10], but can also affect cardiopulmonary function [11,12].

Uncertainties about health effect-relevant PM characteristics and components and their respective sources seriously complicate the process of PM health risk assessment and standard setting as well as the application of cost-effective emission and risk control measures. Whether ambient PM with different compositions and source contributions will have different biological activity and toxicity remains to be determined; this is of substantial importance, both from the scientific and regulatory point of view. Therefore, there is an urgent need for studies to establish the source-composition-effect relationship of PM. Hybrid studies between the toxicology, epidemiology and air quality disciplines may be challenging in this respect. In order to improve the scientific database on this aspect, several European projects have been launched to investigate both the effects of ambient PM collected in European metropolitan as well as rural areas and the relation between toxicity and PM composition and sources (e.g. traffic). These projects use the intratracheal instillation method and focus on the differences between the accumulation or so-called fine mode (aerodynamic diameter 0.1 – 2.5 μm) and the coarse mode (aerodynamic diameter 2.5 – 10 μm) of PM. The studies of Schins et al [10] and Hetland et al [13] have given a first indication that coarse mode particles might be more potent *in vivo *than a fine mode fraction.

Previously, we had investigated the time course of urban PM [7] over a 7-days period, i.e. 2, 4, and 7 days after a single exposure to PM. Gene expression levels and the corresponding products involved in pulmonary and cardiovascular problems were studied in rats with existing pulmonary inflammation. Since the strongest impact on biological effect indicators such as macrophage inflammatory protein-2 (MIP-2), tumor necrosis factor (TNF)-α, endothelin-1 (ET-1) and fibrinogen was observed at 2-days post-exposure, the question arises whether or not significant effects can be detected in less than two days after a single exposure. Indeed, changes in the lung levels of inflammation-associated cytokines have been reported within 2–6 hr after a single exposure to PM or ozone, whereas inflammation, as shown by increased neutrophil levels, often peaks around 24 hr [14-16]. Using residual oil fly ash (ROFA) as a surrogate for ambient PM, Watkinson et al [17] and Campen et al [18] have shown that ROFA induced both immediate (0–6 hr post-instillation) and delayed (24–84 hr) cardiac as well as thermoregulatory responses. A rapid expression of genes after a single PM exposure has also been demonstrated in spontaneously hypertensive (SH) rats [19].

The objective of this study was to optimize the design for *in vivo *PM toxicity screening studies in terms of dose and the time between a single intratracheal exposure and determination of the biological responses. Therefore, in the present study we would gain insight in an optimal dose at which biological effects are still detectable given the relatively small number of observations. Moreover, the time course of effects after a single PM dose was explored. The results should provide important information for the design of upcoming studies in SH rats like the HEPMEAP and PAMCHAR *in vivo *studies in which various ambient PM samples collected across Europe will be compared for their relative toxic potency.

## Results

The results of the body and organ weights, as well as BALF and blood analyses of SH rats exposed to saline or PM (road tunnel dust (RTD) or EHC-93) are given separately for each examined post-exposure time (4, 24 and 48 hr) in additional files (see additional files 1, 2 and 3 respectively).

### Body and organ weights

Body and heart weights did not change in response to RTD or EHC-93 exposure. Conversely, wet lung weights and lung to body weight ratio significantly increased upon exposure to EHC-93 (10 mg/kg) 24 hr after exposure.

### Bronchoalveolar lavage fluid (BALF) analysis

#### Cell profile

**Figure 1:**
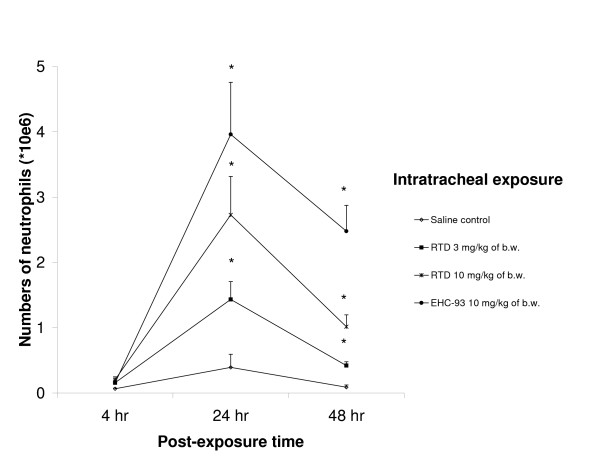
**Neutrophil numbers in BALF measured 24 and 48 hours after exposure. **SH rats were exposed to saline or particulate matter (motorway tunnel dust (RTD) or EHC-93) by a single intratracheal instillation. Values are shown as means ± SEM, *N *= 8–10 and *P < 0.05 significantly different from saline control.

Total cell numbers and neutrophils (Figure 1) were significantly increased in RTD and EHC-93 exposed animals 24 and 48 hr after exposure especially at the highest dose levels. The aforementioned also holds for the number of macrophages with the exception of the response to RTD at 48 hr. Decreased macrophage numbers were observed 4 hr post-exposure to RTD (3 mg/kg). In addition, exposure to EHC-93 resulted in increased BALF lymphocytes numbers at 24 hr (see additional file 2) and no differences in BALF eosinophil numbers were observed. Furthermore, an increase in the percentage of viable BALF cells was seen after exposure to the highest PM doses of RTD and to EHC-93 at 24 and 48 hr post-exposure. For the percentage of viable cells 24 hr after RTD exposure a clear dose-effect relationship was observed, this effect diminishing at 48 hr after instillation. No signs of reduction in the percentage of viable cells were noted.

#### Biochemical characterisation

**Figure 2:**
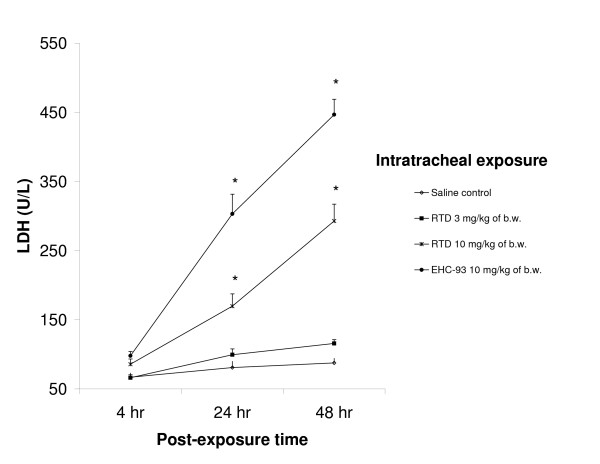
**Lactate dehydrogenase (LDH) activity in BALF measured 24 and 48 hours after exposure. **Legend and significance as indicated in Figure 1.

Since RTD did affect the number of polymorphonuclear neutrophils (PMNs) in BALF, the neutrophil activity marker myeloperoxidase (MPO) was determined for a selected number of BALF samples (see additional file 2). MPO activity was increased at 24 hr post-exposure after exposure to the two highest doses of RTD (3 and 10 mg/kg). Both RTD and EHC-93 induced significantly increased lactate dehydrogenase (LDH) levels in BALF 24 and 48 hr after intratracheal instillation. This increase in cytotoxicity was only observed at the highest dose of 10 mg/kg and the LDH levels increased in time (Figure 2). Exposure to EHC-93 resulted in elevated type II cell damage (alkalin phosphatase (ALP) increase) at all examined time points while RTD caused a dose-dependent damage only at 48 hr after exposure. In addition, elevated concentrations of the macrophage activation marker N-acetyl glucosaminidase (NAG) were found for EHC-93 at all post-exposure times. On the other hand, rats exposed to the low RTD dose of 0.3 mg/kg showed decreased levels of NAG in BALF at 24 and 48 hr. Exposure to this relatively low RTD dose also resulted in decreased BALF protein levels at 24 hr. No additional contrasts were found either for the permeability markers protein and albumin or for the lung damage marker Clara cell protein (CC16) in response to either RTD or EHC-93. Antioxidants levels (reduced glutathione (GSH) and uric acid (UA)) were decreased in rats due to exposure to RTD (1 mg/kg of body weight 24 hr after exposure). Glutathione appeared to be a more sensitive marker since the levels of this antioxidant were affected even by the lowest RTD dose.

#### Cytokine content

**Figure 3:**
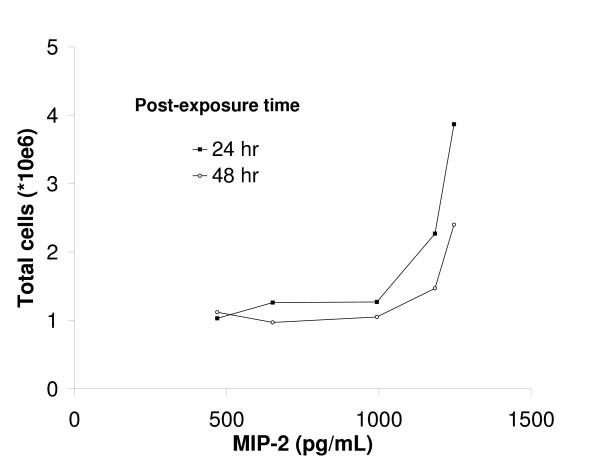
**Correlation between BALF MIP-2 and total cell levels after exposure to different doses of RTD. **The correlation between the MIP-2 concentration at 4 hr and respectively the total cell numbers at 24 and 48 hr post-exposure are shown. Data points for each post-exposure time are saline control followed by the different doses of RTD (respectively 0.3, 1, 3 and 10 mg/kg of body weight). Values are shown as means, *N *= 8–10.

The interleukin-6 (IL-6) concentration was increased already 4 hr after exposure to EHC-93 or the highest dose of RTD. The effect on IL-6 was diminished at a later time for both PM samples but it was still present 48 hr after instillation of EHC-93. A more pronounced response, especially for RTD, was observed for the inflammation marker TNF-α since increased quantities were also seen at lower dose levels (see additional file 1). This effect decreased in the course of time and was still present for the 10 mg/kg of dose of both PM samples 24 hr post-exposure and for RTD even 48 hr after instillation. MIP-2 seemed to be even more sensitive to PM (see additional file 1) as it showed highly significant increases at the lowest dose levels. Again, the peak response was observed 4 hr after exposure but also at 48 hr the highest dose of 10 mg/kg of both PM samples induced a significant MIP-2 response. An increase in BALF MIP-2 concentrations at 4 hr post-exposure coincided with an increase in BALF total cell numbers for the highest RTD doses at 24 as well as 48 hr post-exposure (Figure 3).

### Blood analysis

**Figure 4:**
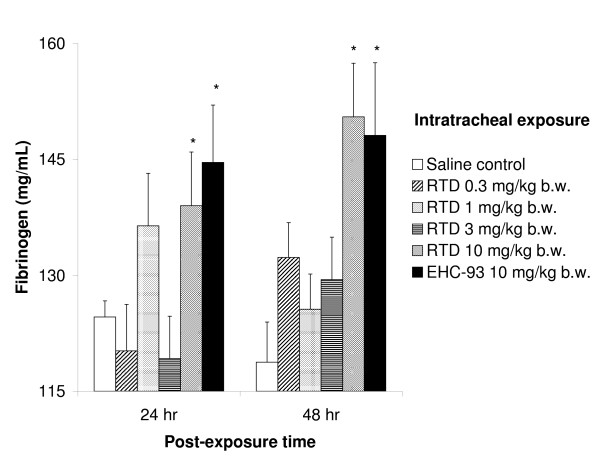
**Fibrinogen levels in blood of SH rats measured 24 and 48 hours after exposure. **Legend and significance as indicated in Figure 1.

Plasma fibrinogen was increased only at 24 and 48 hr after exposure to 10 mg/kg RTD or EHC-93 (Figure 4). No significant changes were found in plasma for big ET-1, the precursor of the vasoconstrictor endothelin-1, neither for the endothelial injury marker von Willebrand factor (vWF). Cell differentials in blood only revealed decreased lymphocyte levels after exposure to the highest dose of RTD and EHC-93, and RTD exposure (10 mg/kg) also resulted in diminished basophilic granulocyte plasma levels, both at 4 hr post-exposure.

### Pulmonary histopathology

**Figure 5:**
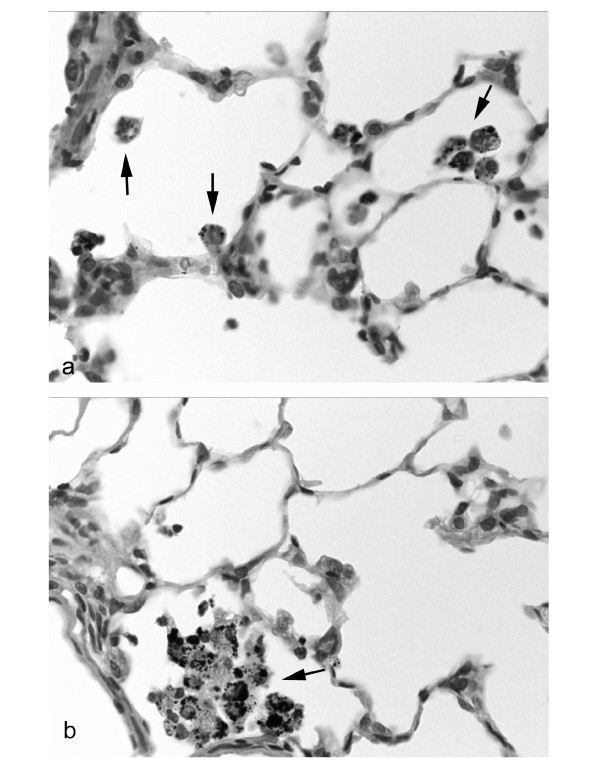
**Lung of SH rat exposed to 3 mg/kg (a) or 10 mg/kg (b) of RTD. **A significant dose-effect relationship is seen for the number of macrophages loaded with PM after exposure to RTD. Slight to moderate quantities of PM are phagocytised by a small number of alveolar macrophages after exposure to the lower RTD dose (a; arrows) while exposure to 10 mg/kg of body weight of RTD resulted in a large number of alveolar macrophages loaded with PM mass (b; arrow). HE, magnification 500×.

Exposure to RTD at doses of 3 and 10 mg/kg of body weight showed a dose-dependent increase in the number of inflammatory foci 24 and 48 hr post-exposure, being significant for the 10 mg/kg of dose at 48 hr (Table 1). On the other hand, exposure to 10 mg/kg of body weight EHC-93 resulted in a more pronounced significant increase in inflammatory foci at both time points. In addition to the observed dose-dependent increase of inflammatory foci, a significant dose-effect relationship was seen for the number of macrophages loaded with PM after exposure to RTD (Figure 5). The high EHC-93 dose also induced a significant increase the number of macrophages in SH rats that phagocytized RTD. The histological evaluation of BrdU-labelling in control SH rats showed besides a normal background labelling and a high labelling in bronchoalveolar lymphoid tissue (BALT) and perivascular lymphoid tissues, a pronounced labelling in the inflammatory foci. Exposure to RTD as well as EHC-93 caused a pronounced increase in the size of the labelled areas of the inflammatory foci (Table 1).

**Table 1:** Summary of histopathological changes in lungs of SH rats exposed to particulate matter or saline.

Post-exposure time		24 hr				48 hr			
Group		1	4	5	6	1	4	5	6
Number examined		9	9	9	9	10	10	10	10
									
Alveolar Mφ	minimal	2	6	3	5	4	6	3	-
	slight	-	2	5	1	1	4	2	6
	moderate	-	1	1	1	-	-	4	2
	marked	-	-	-	-	-	-	-	2
	overall	2	13	16	10	6	14	19	26
									
Inflammatory foci thick septa + alveolar Mφ	minimal	6	4	3	-	6	2	-	-
	slight	3	5	3	3	2	4	-	-
	moderate	-	-	3	3	2	4	7	3
	marked	-	-	-	3	-	-	3	7
	overall	12	14	18	27	16	22	33	37
									
BrdU score	number examined	6	6	6	6	6	6	6	6
	minimal	2	-	-	-	6	1	-	-
	slight	1	1	-	-	-	3	-	-
	moderate	-	3	3	-	-	2	3	2
	marked	-	2	2	3	-	-	3	2
	strong	-	-	1	3	-	-	-	2
	overall	4	19	22	27	6	13	21	24

The lungs of SH rats exposed to saline (group1), 3 or 10 mg/kg of body weight RTD (respectively group 4 and 5) or 10 mg/kg of body weight EHC-93 (group 6) were examined at 24 or 48 hours post-exposure. In the overall score, a weight factor of 1 to 5 is included for the score minimal to strong. Abbreviations: Mφ – macrophages.

## Discussion

In this study, several dose-dependent biological responses were measured after exposure to RTD or EHC-93 by intratracheal instillation at all three post-exposure times (4, 24 and 48 hr). As expected on the basis of previous studies [14-16], the inflammation-associated cytokine response in the lung is most prominent rapidly after exposure while inflammatory cell influx peaks 24 hr post-exposure. No deaths or immediately life-threatening health effects have been observed after a single exposure to PM; this latter aspect is supported by the relatively slight changes in body or lung weight, macroscopic findings noted in necropsy and the overall lung pathology. Furthermore, the marker for cytotoxicity, LDH, is released into BALF only at the highest dose level. The low levels of protein and albumin in the BALF as well as the lack of Clara cell secretory protein CC16 once more indicated that these relatively high doses used for instillation did not induce serious toxicity.

All doses used in the present study, including the lowest dose of 0.3 mg/kg, were above levels which can be expected to occur in realistic human occupational or environmental exposures [20]. On the other hand, these levels are justified considering relative PM toxicity assessment of different types of particles and their underlying biological mechanisms. For most health effect parameters, a clear dose-dependent response pattern is observed. However, the biological responses induced by the highest 10 mg/kg dose of RTD, and especially EHC-93, appeared more extreme and did not seem to fit in the general pattern of this curve. These effects could be attributed to high dose and could be seen as a different response pattern, which is probably caused by overload of the lungs [21,22]. Remarkably, however, is the observation that there are still lung macrophages without phagocytized PM and there are also no signs of loose PM in the alveoli indicating that the dose of 10 mg/kg of body weight did not result in overload of the lungs in the present study (unpublished observations). Although separate screening of size-different PM fractions will occur in several European projects, the present study used a RTD PM sample of pooled coarse and fine fractions to increase the yield of PM toxicity, thereby improving the probability of hitting the biological responses. Furthermore, using combined instead of separate fractions is in accordance with the policy to restrict the number of experimental animals.

PM exposure can provoke pulmonary injury/inflammation, an oxidative stress response as well as systemic effects [7,23-27]. Individuals with existing diseases are more susceptible to PM exposure; exacerbation of pulmonary inflammation in susceptible people might therefore be a central mechanism by which PM exerts its toxicity [28,29]. Since susceptible subjects are more at risk, the spontaneously hypertensive rat was selected as an animal model for assessment of relative PM toxicity. The SH rat has proven to be an animal model representative for subjects with systemic hypertension and has also shown to facilitate measurement of PM toxicity [30]. In the present study, pulmonary injury was determined by several parameters measured in BALF (LDH, ALP, protein, albumin, and CC16) in addition to macroscopic and microscopic pathology of the lung. Gross cellular lung damage is absent even after exposure to very high PM doses despite the high neutrophilia in PM exposed lungs. Such an inflammatory reaction in the lung in the absence of serious damage has also been reported in rats after exposure to relatively low PM dose (~0.6 – 1 mg/kg) measured 18 hr post instillation [10].

The present PM exposures resulted in a rapid response of pro-inflammatory mediators (MIP-2, TNF-α, and IL-6). MIP-2 plays a key role in inflammatory cell recruitment since MIP-2 levels have often preceded the increases in neutrophils [14,31]. TNF-α has been suggested to be mostly released in connection to increased neutrophils though it is released by macrophages. In addition, IL-6 levels would rise when TNF-α becomes down-regulated [32]. This appears to be true also for PM induced inflammation shown here. Pulmonary inflammation due to PM exposure is evident in the present study since the neutrophil numbers in BALF increased tremendously after exposure to the highest RTD doses or EHC-93 and some neutrophil influx was already seen at a low PM dose 4 hr after exposure. In spite of this, only the high RTD doses of 3 and 10 mg/kg of body weight have also shown induced neutrophil activity as indicated by increased MPO activity. Increased BALF neutrophil numbers, and by that pulmonary inflammation is a consistent PM effect that has also been reported in other rat instillation studies [10,15]. Elevated numbers of macrophages, as observed in the present study, have not consistently been shown, which is probably caused by variability in the lavage procedure. However, a clear macrophage increase is shown in humans 18 hr after exposure to diesel exhaust [33]. The finding of reduced macrophage numbers in BALF 4 hr after exposure to 3 mg/kg of body weight of RTD most likely represents an increased macrophage adherence to the epithelium in a relatively early phase after exposure. Later onwards the macrophage numbers increase due to the demand for particle clearance. The importance of lymphocytes in the induction of an early inflammatory response by PM exposure has been suggested earlier for diesel exhaust [2,3,34] and exposure to EHC-93 resulted in increased BALF lymphocyte numbers, which supported this finding. It is obvious that in the normal resting unchallenged state there are a certain percentage of aged cells with reduced membrane integrity. Exposure resulted in a relatively massive recruitment and influx of inflammatory cells, which are far more viable. This means that it is not the viability itself which has increased, but rather an influx of fresh and lively cells. In addition, 24 hr after exposure to low doses of RTD decreased levels of the antioxidants GSH and UA were observed. This implies that oxidative stress will also play a role in the mechanisms that will lead to adverse health effects, which should be investigated more thoroughly in future studies.

Systemic changes following inflammatory reactions or other immune responses in the lung are likely. A decrease in the number of blood lymphocytes is one example of a possible systemic change. In the present study, this was only observed shortly after PM exposure in the absence of pulmonary injury and when pulmonary inflammation was not yet obvious. A small decrease in circulating lymphocytes has been demonstrated before as a result of PM exposure by intratracheal instillation and/or inhalation in both rats [23,35] and humans [36]. An increase in circulating neutrophils coincided with a decrease in lymphocytes upon exposure by inhalation [35,36] while instillation studies, including the present study, revealed no increase in blood neutrophils [23]. Although the biological significance and impact remains unclear, the decrease in lymphocytes is consistent in these studies. In the present study, reduction in blood lymphocytes preceded the increase in BALF lymphocytes, which could be explained by increased adherence of lymphocytes to the pulmonary vasculature, leading to subsequent lymphocyte influx to the airways. Another systemic effect marker, fibrinogen, which is a risk factor for cardiovascular disease [37,38], contributes to plasma viscosity as does vWF, a pro-coagulant product of the endothelium [39]. Exposure to a high PM dose resulted in increased plasma fibrinogen but no changes were observed in plasma vWF levels. Various effects of pollutant particles on blood fibrinogen are reported in the literature. Elevation, reduction as well as no changes in fibrinogen levels have been described in either humans [40-42] or animals [43,44] upon exposure to PM under variable conditions. These contradicting results could be due to differences in the source contribution, PM dose or differences in exposure pathways, time of evaluation, and/or variability in response within groups. Given that vWF also contributes to plasma viscosity the observed steady vWF levels were unexpected and could be attributed to a stronger effect of fibrinogen on viscosity [39]. However, the unchanged vWF plasma levels indicate that exposure to RTD and EHC-93 in these concentrations did not result in endothelial damage.

## Conclusion

In the present study, we have shown that the optimal dose for determining the toxicity ranking of ambient-derived PM samples appeared to be between 3 and 10 mg PM/kg of body weight. This is valid for the applied study design in spontaneously hypertensive rats. This is supported by the fact that, in the range of these doses, biological responses such as pulmonary inflammation, oxidative stress and effects observed in the circulation can be detected without the presence of pulmonary injury or toxicity. At lower doses only some inflammatory effects can be detected, probably too few to be able to discriminate between PM samples while a completely different response pattern was observed with the highest dose. Most likely, additional effects could be found at lower doses by increasing the number of animals but searching for an optimal dose using small animal numbers was an argument behind this study. In addition to the dose, 24 hr post-exposure seemed to be the appropriate time to assess PM toxicity since the majority of the health effects are observed one day after PM exposure compared to the other times examined. The aforementioned considerations provide a good basis for conducting *in vivo *PM toxicity screening studies in spontaneously hypertensive rats.

## Methods

### Animals

Spontaneously hypertensive male rats (SHR/NHsd) of 11–12 weeks old and weighing 250–350 g were obtained from the breeding colony of Harlan (Indianapolis, Indiana, USA). Animals were housed in macrolon cages Type 3 in a room with HEPA-filtered air and a constant climate (room temperature 21 ± 2°C and relative humidity 40–70%) with a 12-hr light/dark cycle (light on at 8.00 a.m.). Special rodent food (SSP-TOX standard/eromix pellets 10 mm non-radiated; Hope Farms, Woerden, the Netherlands) and tap water via an automatic drinking-water system was supplied *ad libitum*. Immediately after arrival, the animals were weighed and randomly allocated into exposure groups. Experiments started after an acclimatisation period of at least 7 days. Experiments were approved by the Ethical Review Committee of the National Institute for Public Health and the Environment.

### Objectives and study design

The objective of the present study is to determine the optimal dose of and time after a single exposure to PM at which in subsequent studies various PM samples collected throughout Europe can be tested and ranked for their toxic potency. It is hypothesized that a single exposure to ambient particulate matter in spontaneously hypertensive rats will lead to an immediate pulmonary injury/inflammation, antioxidant depletion. This may consequently be followed by alterations in markers which are symptomatic for changes in blood coagulation factors and circulating blood cell populations and might eventually affect cardiovascular function. In order to address the hypothesis, rats were exposed to various doses of PM collected from ambient air near a motorway tunnel using high-volume techniques. In addition, a reference sample (EHC-93, see below) has been used to allow inter study comparison. The biological responses to these PM samples were determined after three distinct time points: 4, 24 or 48 hr post-exposure. In order to increase the sensitivity of the analysis, spontaneously hypertensive (SH) rats were selected, as being representative for people with systemic hypertension [30]. A wide selection of markers for toxicity and inflammation were examined.

### Sampling and characterisation of particles

PM was collected on polyurethane foam (PUF) at the exit of a motorway tunnel in Hendrik-Ido-Ambacht (HIA) in the Netherlands using a high volume cascade impactor (HVCI) [45]. Coarse (2.5–10 μm) and fine (0.1–2.5 μm) PM were sampled separately on PUF. The PUF was cleaned before use in 3 successive baths with 30 min sonications, in water, in ethanol and in methanol respectively. The methanol extraction of collected PM from PUF includes potentially both advantages and disadvantages. Methanol, unlike water, wets the porous structure of PUF and a high extraction efficiency (up to 90–95%) of the collected PM mass has been shown [46]. The method has already extensively been applied in several EU funded projects [46-48]. After washing, PUF was dried at 50°C overnight and weighed. The collected PM samples were extracted from the PUF into 100% methanol by sonicating 2 or 3 times for 30 min, each time adding new methanol to increase PM yield. Subsequently, methanol was removed by evaporation at 30°C overnight to yield dry PM samples. Coarse and fine PM fractions, collected during week 7 and 10 in 2002, were pooled in this study to represent an integrated PM sample from the motorway tunnel at HIA (road tunnel dust, RTD). An urban air PM sample (Ottawa dust; EHC-93) recovered by vacuuming of bag-house filters of the Environmental Health Centre in Ottawa in Canada was used in this study as a second ambient PM sample. The chemical composition and biological reactivity of EHC-93 have been described earlier [49,50]. In addition, the chemical composition of both RTD and EHC-93 was determined in the present study and the outcomes are shown in Table 2.

**Table 2:** Chemical composition of RTD and EHC-93 (μg/g).

	RTD	EHC-93
Cd	7.4	18.3
Pb	176	5700
Mg	5207	15983
Al	5184	14562
Si	10409	43713
Ca	11738	123403
V	83.2	99.1
Cr	53.3	44.9
Mn	376	445
Fe	12929	17759
Ni	70	41
Cu	532	760
Zn	2678	10370
Na	50819	22339
K	3172	6436
NH4	42868	222
Cl	79679	23916
NO3	130374	24319
SO4	47355	56664
PAH	0.66	0.86

### Exposure by intratracheal instillation

Table 3 shows the exposure material and doses of the different animal groups (*N *= 10/group) used in this study. Rats were exposed to RTD suspended in saline (0.15, 0.5, 1.5 or 5 mg/mL) after anaesthesia with 4% halothane (Ceva, Maassluis, the Netherlands) and instilled with a volume of 2 mL/kg of body weight resulting in 0.3, 1, 3 or 10 mg/kg of body weight. EHC-93 (5 mg/mL) was employed as a reference sample used in previous studies [7,51,52]. Briefly, after a few min of 4% halothane anaesthesia the rat was fixed with the forelegs on a small vertical (60 degrees) table. The neck was lighted and a cannula was inserted through the mouth into the trachea just above the bifurcation. Proper placement of the cannula was checked with a 10 mL glass syringe BD cornwall 2193F (Becton Dickinson, Grenoble, France). With the same syringe hyperventilation was induced to get a period of about 3 seconds without breathing of the animal. During this period the volume of saline or PM sample was instilled through the cannula with a long needle ending just above the end of the trachea cannula. Immediately after the instillation, the glass syringe was used again a few times to inflate the lungs and spread the instilled volume over the lungs.

**Table 3:** Exposure and dose of experimental animal groups (*N *= 10/group).

Group	Exposure	Dose (mg/kg of body weight)
1	Saline	-
2	RTD	0.3
3	RTD	1
4	RTD	3
5	RTD	10
6	EHC -93	10

Biological responses were examined 4, 24 and 48 hours after exposure. Abbreviations: RTD – particulate matter collected at the motorway tunnel in Hendrik-Ido-Ambacht, the Netherlands; EHC-93 – Ottawa dust [22].

### Necropsy

Two hours before necropsy, the animals (*N *= 6/group) were subcutaneously (s.c.) injected with 40 mg/kg of body weight 5-bromo-2-deoxyuridine (20 mg/mL BrdU; Sigma-Aldrich, Zwijndrecht, the Netherlands). At necropsy, animals were anaesthetised with a mixture of Ketamine/Rompun (1 mL/kg body weight i.p. of a 10:4 mix of 100 mg/mL Ketamine (Aesculaap, Boxtel, the Netherlands) and 20 mg/ml Rompun (Bayer, Leverkusen, Germany)) and sacrificed by exsanguination via the abdominal aorta. Necropsy was performed at 4, 24 or 48 hr post-exposure. Saline perfusion of the lungs was performed via the right cardiac ventriculum only for those animals that did not received BrdU (*N *= 4/group). In this study, the right lung was used to obtain BALF after ligation of the left bronchus. The right lung was lavaged (three in- and out lavages using same fluid) with a volume of saline corresponding with 27 mL/kg body weight at 37°C. The recovered BALF was placed on ice. The left lung was dissected, weighed, and preserved for histopathology after fixation for one hour under a constant pressure of 20-cm H_2_O with 10% phosphate buffered formalin.

### Histopathology

The left lung was embedded in paraplast and 5 μm thick lung sections from animals exposed to saline, 3 or 10 mg/kg RTD, and 10 mg/kg EHC-93 (experimental groups 1 and 4–6; Table 3) collected 24 and 48 hr post-exposure were stained with hematoxylin-eosin (HE). The pathological lesions were semiquantitatively, blindly scored by light microscopy (minimal, slight, moderate, marked and strong; *N *= 9–10/group). Furthermore, the cumulative cell proliferation was examined by immunohistochemical staining with an anti-BrdU antibody (Boehringer, Mannheim, Germany) labelled with peroxidase. BrdU-labelling was also semiquantitatively scored by estimation of inflammatory areas with higher labelling index (*N *= 6/group).

### BALF analysis

The BALF from each animal was centrifuged at 400 g, 4°C, for 10 min. The cell-free fluid from the lavage was used for measurements of cellular toxicity, inflammation and oxidative stress. The pellet from the lavage was resuspended in 1 mL saline and used for total cell counts, preparation of cytospins for differential cell counts and measurement of cell viability.

#### Cell counts and differentials

Total cell number was determined by mixing 0.5 mL of the cell suspension with 9.5 mL Isoton II (Beckman Coulter B.V., Mijdrecht, the Netherlands) and subsequently counting in a Coulter Counter Z1 and/or Z2 (Beckman Coulter B.V.). Cytospin slides were made in duplicate for differential cell counts and stained according to May-Grünwald and Giemsa. Per cytospin slide, 200 cells were counted (total of 400 cells per exposure) and the proportion of each cell type (macrophages, neutrophilic granulocytes, eosinophilic granulocytes and lymphocytes) was calculated on the basis of total cells per BALF sample. The viability of BALF cells was only examined 24 and 48 hr after exposure. Cell suspensions were diluted 1:1 with 0.2% trypan blue and the numbers of living and dead cells were counted in a Bürker counting chamber.

#### Biochemistry

The neutrophil activity marker MPO was assayed. Briefly, samples were diluted 1:5 in freshly prepared assay solution (0.01 M sodium phosphate buffer pH 7.0, 0.01 M H_2_O_2 _and 0.015 M guaiacol (Sigma-Aldrich)). The generation of tetra-guaiacol was measured at 470 nm for 2 min at 37°C at intervals of 15 seconds and and the change of optical density (OD) per min was calculated from the initial rate. The MPO activity was then calculated from the formula: U/mL = ΔOD/min × 0.752. One unit of the enzyme is defined as the amount that consumes 1 μmol H_2_O_2 _per min [53]. LDH, NAG, ALP, UA and albumin were determined using a commercially obtained reagent kit (Roche Nederland B.V, Almere, the Netherlands). Total protein was determined using a reagent kit obtained from Pierce (Etten-Leur, the Netherlands). Enzyme-linked immunosorbent assay (ELISA) was used to determine CC16 in BALF as described before [54]. Briefly, high-binding microtiterplates were coated with anti-CC16 serum diluted 1:4,000 in coating buffer (50 mM carbonate pH 9.4). Between each incubation step the plates were washed four times in washing buffer (PBST; phosphate buffered saline (PBS) with 0.1% Tween 20. The CC16 standards (10, 3.33, 1.11, 0.37, 0.123, 0.041, 0.0137 and 0 ng/mL) diluted in assay buffer (PBST with 0.5% BSA and the BALF samples diluted 1:10,000 were added in duplicate and incubated at 37°C for 90 min. Subsequently, plates were incubated with a polyclonal anti-CC16 antibody (1:2,000 diluted in assay buffer) at 37°C for 90 min followed by an incubation with 0.10 mL streptavidin HRP (1:10,000 diluted in assay buffer) for 30 min at room temperature. TMB-reagens was added for colour development and after stopping the reaction by adding H_2_SO_4_, the absorbance was measured at 450 nm. The CC16 concentrations in BALF samples were calculated on the basis of absorbance readings of the standards. Glutathione (GSH and GSSG) was measured as described previously [54]. Quantification of GSSG was accomplished by conjugation of GSH with 2-vinylpyridine in ethanol (1:20 ratio v/v). Both the vinylpyridine-treated and untreated samples were assayed for GSH concentrations by an enzymatic recycling method using glutathione reductase at 415 nm for 2 min. GSSG was calculated from the vinylpyridine-treated sample (GSSG = 2 × GSH) and GSH from the untreated sample (GSH = total glutathione - GSSG × 2). LDH was measured as a marker for cytotoxicity, NAG as an indicator for macrophage activation, ALP as a marker for type II cell damage. Albumin and total protein levels were measured as indicators for increased permeability of the alveolar-capillary barrier and CC16 for lung cell damage. In addition, the antioxidants uric acid and glutathione were measured as markers for oxidative stress. The inflammatory mediators IL-6, TNF-α and MIP-2 were determined using commercially available ELISA kit (Biosource, Etten-Leur, the Netherlands).

### Blood analysis

Fibrinogen, a marker of the coagulation response, was determined in citrate plasma (DiaFibrinogen kit Cat. no. 305100; DiaMed Benelux n.v., Turnhout, Belgium). The marker for early endothelial injury, vWF, was determined in citrate plasma by ELISA (Kordia B.V., Leiden, the Netherlands) using pooled citrate plasma of unexposed rats to prepare a reference standard. Cell differentials were determined in EDTA(K_3_) (Terumo Europe N.V., Leuven, Belgium) anticoagulated blood using an H1-E Multi Species Haematology Analyser (Bayer B.V., Mijdrecht, the Netherlands). The following parameters were measured: white and red blood cell concentrations, haemoglobin and platelet concentrations, the number of lymphocytes, monocytes, eosinophilic and basophilic granulocytes, as well as the haematocrit value. The ET-1 precursor bigET-1 was measured by ELISA (IBL, Hamburg, Germany) only 24 and 48 hr post-exposure.

### Statistical analysis

All biological effect parameters were log-transformed and, subsequently, a two-way analysis of variance (ANOVA) was performed. Two-way ANOVA techniques were used to assess differences due to PM (RTD or EHC-93) exposure, day-to-day variation (caused by different necropsy days (*N *= 5) for a specific post-exposure time) and their interaction. The above-mentioned differences were analysed for all three post-exposure times (4, 24 and 48 hr) separately and Bonferroni was used for post-hoc analyses. In case log-transformation did not result in normal distribution of the measured parameter the non-parametric test Kruskal-Wallis rank sum test (used for the following markers: BALF lymphocytes and eosinophils; viability; protein, albumin, IL-6, MIP-2, and TNF-α at 4 hr; NAG, glutathione, GSSG, GSH, GSSG:GSH ratio, and UA at 4 as well as 24 hr post-exposure; fibrinogen; blood lymphocytes; basophilic granulocytes) or Wilcoxon signed-rank test (MPO) was performed to reveal differences between specific groups. The values are expressed as means ± standard error of the mean (SEM) and *P *values <0.05 were regarded significant. All above-mentioned statistical analysis was performed using S-Plus software (MathSoft, Inc). The histological parameters were statistically tested with the non-parametric Wilcoxon test.

## List of abbreviations

ANOVA – analysis of variance; ALP – alkalin phosphatase; BALF – bronchoalveolar lavage fluid; BALT – bronchoalveolar lymphoid tissue; BrdU – 5-bromo-2-deoxyuridine; b.w. – body weight; CC16 – Clara cell protein; EHC-93 – urban PM sample, Ottawa dust; ELISA – enzyme-linked immunosorbent assay; ET-1 – endothelin-1; GSH – reduced glutathione; GSSG – oxidized glutathione; HE – hematoxylin-eosin; HIA- Hendrik-Ido-Ambacht; HVCI – high volume cascade impactor; IL-6 – interleukin-6; LDH – lactate dehydrogenase; MIP-2 – macrophage inflammatory protein-2; MPO – myeloperoxidase; Mφ – macrophages; NAG – N-acetyl glucosaminidase; OD – optical density; PM – particulate matter; PBS – phosphate buffered saline; PBST – PBS with 10 mM 0.1% Tween 20; PMNs – polymorphonuclear neutrophils; PUF – polyurethane foam; ROFA – residual oil fly ash; RTD – road tunnel dust; s.c. – subcutaneous; SEM – standard error of the mean; SH – spontaneously hypertensive; TNF-α – tumor necrosis factor α; UA – uric acid; vWF – von Willebrand factor

## Competing interests

The author(s) declare that they have no competing interests.

## Authors' contributions

MEG has designed, coordinated and supervised the experimental work of this study, took part in the autopsies including the collection of BALF samples, processed the data including tables and figures, carried out the statistical analysis, and interpret the results and drafted the manuscript.

AJFB participated in the design and coordination of the study, carried out the *in vivo *experiments including sample handling, and participated in the statistical analysis.

DLACL participated in the design, supported the *in vivo *experiments and collection of blood and tissue samples, carried out several BALF and blood analysis.

JAMAD supported collection of lung tissue, performed histopathology and the statistical analysis for this part of the study.

TS is overall co-ordinator of the HEPMEAP project and participated in the design of the study and interpretation of the data.

ROS is overall co-ordinator of the PAMCHAR project and participated in the design of the study and interpretation of the data.

LvB is RIVM co-ordinator of the HEPMEAP project and participated in the design of the study and interpretation of the results.

FRC is RIVM co-ordinator of the PAMCHAR project, participated in conceiving the study, its design, interpretation of the results and is co-writer of the manuscript.

All authors have read, reviewed, commented and approved the final manuscript.

## Supplementary Material

Additional File 1 **Results of the body and organ weights, BALF and blood analyses measured 4 hours post-exposure.** Analyses were performed on material from particulate matter or saline exposed SH rats. Values are shown as means and 95% confidence interval (CI). P values < 0.05 were regarded significant compared to saline. *P < 0.05, **P < 0.01 and ***P < 0.001. Click here for file

Additional File 2 **Results of the body and organ weights, BALF and blood analyses measured 24 hours post-exposure.** Legend and significance as indicated in additional file 1. Click here for file

Additional File 3 **Results of the body and organ weights, BALF and blood analyses measured 48 hours post-exposure.** Legend and significance as indicated in additional file 1. Click here for file

## References

[B1] Bell ML, Samet JM, Dominici F (2004). Time-series studies of particulate matter. Annu Rev Public Health.

[B2] Salvi SS, Nordenhäll C, Blomberg A, Rudell B, Pourazar J, Kelly FJ, Sandström T (2000). Acute exposure to diesel exhaust increases IL-8 and GRO-alpha production in healthy human airways. Am J Respir Crit Care Med.

[B3] Stenfors N, Nordenhall C, Salvi SS, Mudway I, Soderberg M, Blomberg A, Helleday R, Levin JO, Holgate ST, Kelly FJ, Frew AJ, Sandström T (2004). Different airway inflammatory responses in asthmatic and healthy humans exposed to diesel. Eur Respir J.

[B4] Lemos M, Lichtenfels AJ, Amaro JE, Macchione M, Martins MA, King M, Bohm GM, Saldiva PH (1994). Quantitative pathology of nasal passages in rats exposed to urban levels of air pollution. Environ Res.

[B5] Gulisano M, Marceddu S, Barbaro A, Pacini A, Buiatti E, Martini A, Pacini P (1997). Damage to the nasopharyngeal mucosa induced by current levels of urban air pollution: a field study in lambs. Eur Respir J.

[B6] Calderon-Garciduenas L, Roy-Ocotla G (1993). Nasal cytology in southwest metropolitan Mexico City inhabitants: a pilot intervention study. Environ Health Perspect.

[B7] Ulrich MM, Alink GM, Kumarathasan P, Vincent R, Boere AJ, Cassee FR (2002). Health effects and time course of particulate matter on the cardiopulmonary system in rats with lung inflammation. J Toxicol Environ Health A.

[B8] Adamson IY, Prieditis H, Vincent R (1999). Pulmonary toxicity of an atmospheric particulate sample is due to the soluble fraction. Toxicol Appl Pharmacol.

[B9] Li XY, Gilmour PS, Donaldson K, MacNee W (1997). In vivo and in vitro proinflammatory effects of particulate air pollution (PM10). Environ Health Perspect.

[B10] Schins RP, Lightbody JH, Borm PJ, Shi T, Donaldson K, Stone V (2004). Inflammatory effects of coarse and fine particulate matter in relation to chemical and biological constituents. Toxicol Appl Pharmacol.

[B11] Wichers LB, Nolan JP, Winsett DW, Ledbetter AD, Kodavanti UP, Schladweiler MC, Costa DL, Watkinson WP (2004). Effects of instilled combustion-derived particles in spontaneously hypertensive rats. Part I: Cardiovascular responses. Inhal Toxicol.

[B12] Wichers LB, Nolan JP, Winsett DW, Ledbetter AD, Kodavanti UP, Schladweiler MC, Costa DL, Watkinson WP (2004). Effects of instilled combustion-derived particles in spontaneously hypertensive rats. Part II: Pulmonary responses. Inhal Toxicol.

[B13] Hetland RB, Cassee FR, Refsnes M, Schwarze PE, Lag M, Boere AJ, Dybing E (2004). Release of inflammatory cytokines, cell toxicity and apoptosis in epithelial lung cells after exposure to ambient air particles of different size fractions. Toxicol In Vitro.

[B14] Driscoll KE, Simpson L, Carter J, Hassenbein D, Leikauf GD (1993). Ozone inhalation stimulates expression of a neutrophil chemotactic protein, macrophage inflammatory protein 2. Toxicol Appl Pharmacol.

[B15] Kodavanti UP, Schladweiler MC, Richards JR, Costa DL (2001). Acute lung injury from intratracheal exposure to fugitive residual oil fly ash and its constituent metals in normo- and spontaneously hypertensive rats. Inhal Toxicol.

[B16] Kodavanti UP, Jaskot RH, Costa DL, Dreher KLRA (1997). Pulmonary proinflammatory gene induction following acute exposure to residual oil fly ash: roles of particle associated metals. Inhal Toxicol.

[B17] Watkinson WP, Campen MJ, Costa DL (1998). Cardiac arrhythmia induction after exposure to residual oil fly ash particles in a rodent model of pulmonary hypertension. Toxicol Sci.

[B18] Campen MJ, Costa DL, Watkinson WP (2000). Cardiac and thermoregulatory toxicity of residual oil fly ash in cardiopulmonary-compromised rats. Inhal Toxicol.

[B19] Kooter IM, Pennings JLA, Opperhuizen A, Cassee FR (2005). Gene expression pattern in spontaneously hypertensive rats exposed to urban particulate matter (EHC-93). Inhal Toxicol.

[B20] Oberdorster G, Yu CP (1999). Lung dosimetry – considerations for noninhalation studies. Exp Lung Res.

[B21] Morrow PE (1988). Possible mechanisms to explain dust overloading of the lungs. Fundam Appl Toxicol.

[B22] Oberdorster G (1995). Lung particle overload: implications for occupational exposures to particles. Regul Toxicol Pharmacol.

[B23] Kodavanti UP, Schladweiler MC, Ledbetter AD, Hauser R, Christiani DC, McGee J, Richards JR, Costa DL (2002). Temporal association between pulmonary and systemic effects of particulate matter in healthy and cardiovascular compromised rats. J Toxicol Environ Health A.

[B24] Donaldson K, Stone V, Borm PJ, Jimenez LA, Gilmour PS, Schins RP, Knaapen AM, Rahman I, Faux SP, Brown DM, MacNee W (2003). Oxidative stress and calcium signaling in the adverse effects of environmental particles (PM10). Free Radic Biol Med.

[B25] Brook RD, Brook JR, Rajagopalan S (2003). Air pollution: the "Heart" of the problem. Curr Hypertens Rep.

[B26] Handzel ZT (2000). Effects of environmental pollutants on airways, allergic inflammation, and the immune response. Rev Environ Health.

[B27] Peden DB (2001). Air pollution in asthma: effect of pollutants on airway inflammation. Ann Allergy Asthma Immunol.

[B28] Bateson TF, Schwartz J (2004). Who is sensitive to the effects of particulate air pollution on mortality? A case-crossover analysis of effect modifiers. Epidemiology.

[B29] Tao F, Gonzalez-Flecha B, Kobzik L (2003). Reactive oxygen species in pulmonary inflammation by ambient particulates. Free Radic Biol Med.

[B30] Kodavanti UP, Schladweiler MC, Ledbetter AD, Watkinson WP, Campen MJ, Winsett DW, Richards JR, Crissman KM, Hatch GE, Costa DL (2000). The spontaneously hypertensive rat as a model of human cardiovascular disease: evidence of exacerbated cardiopulmonary injury and oxidative stress from inhaled emission particulate matter. Toxicol Appl Pharmacol.

[B31] Driscoll KE, Hassenbein DG, Carter J, Poynter J, Asquith TN, Grant RA, Whitten J, Purdon MP, Takigiku R (1993). Macrophage inflammatory proteins 1 and 2: expression by rat alveolar macrophages, fibroblasts, and epithelial cells and in rat lung after mineral dust exposure. Am J Respir Cell Mol Biol.

[B32] Wardle EN (1993). Cytokines: an overview. Eur J Med.

[B33] Rudell B, Blomberg A, Helleday R, Ledin M-C, Lundbäck, Stjernberg N, Hörstedt P, Sandström T (1999). Bronchoalveolar inflammation after exposure to diesel exhaust: comparison between unfiltered and particle trap filtered exhaust. Occup Environ Med.

[B34] Fujimaki H, Ui N, Endo T (2001). Induction of inflammatory response of mice exposed to diesel exhaust is modulated by CD4^+ ^and CD8^+ ^T cells. Am J Respir Crit Care Med.

[B35] Gordon T, Nadziejko C, Schlesinger R, Chen LC (1998). Pulmonary and cardiovascular effects of acute exposure to concentrated ambient particulate matter in rats. Toxicol Lett.

[B36] Salvi S, Blomberg A, Rudell B, Kelly F, Sandstrom T, Holgate ST, Frew A (1999). Acute inflammatory responses in the airways and peripheral blood after short-term exposure to diesel exhaust in healthy human volunteers. Am J Respir Crit Care Med.

[B37] Lowe GD, Lee AJ, Rumley A, Price JF, Fowkes FG (1997). Blood viscosity and risk of cardiovascular events: the Edinburgh Artery Study. Br J Haematol.

[B38] Ernst E (1993). The role of fibrinogen as a cardiovascular risk factor. Atherosclerosis.

[B39] Blann A, Bignell A, McCollum C (1998). von Willebrand factor, fibrinogen and other plasma proteins as determinants of plasma viscosity. Atherosclerosis.

[B40] Seaton A, Soutar A, Crawford V, Elton R, McNerlan S, Cherrie J, Watt M, Agius R, Stout R (1999). Particulate air pollution and the blood. Thorax.

[B41] Pekkanen J, Brunner EJ, Anderson HR, Tiittanen P, Atkinson RW (2000). Daily concentrations of air pollution and plasma fibrinogen in London. Occup Environ Med.

[B42] Peters A, Doring A, Wichmann HE, Koenig W (1997). Increased plasma viscosity during an air pollution episode: a link to mortality?. Lancet.

[B43] Gardner SY, Lehmann JR, Costa DL (2000). Oil fly ash-induced elevation of plasma fibrinogen levels in rats. Toxicol Sci.

[B44] Nadziejko C, Fang K, Chen LC, Cohen B, Karpatkin M, Nadas A (2002). Effect of concentrated ambient particulate matter on blood coagulation parameters in rats. Res Rep Health Eff Inst.

[B45] Demokritou P, Kavouras IG, Ferguson ST, Koutrakis P (2002). Development of a high volume cascade impactor for toxicological and chemical characterization studies. Aerosol Science and Technology.

[B46] Jalava P, Salonen RO, Hälinen AI, Sillanpää M, Sandell E, Hirvonen M-R (2005). Effects of sample preparation on chemistry, cytotoxicity, and inflammatory responses induced by air particulate matter. Inhal Toxicol.

[B47] Salonen RO, Pennanen AS, HäIinen AI, Hirvonen M-R, Sillanpää M, Hillamo R, Karlsson V, Koskentalo T, Aarnio P, Ferguson S, Koutrakis P (2000). A chemical and toxicological comparison of urban air PM_10 _collected during winter and spring in Finland. Inhal Toxicol.

[B48] Cassee FR, Fokkens PHB, Leseman DLAC, Bloemen HJTh, Boere AJF (2003). Respiratory allergy and inflammation due to ambient particles (RAIAP) collection of particulate matter samples from 5 European sites with high volume cascade impactors. RIVM report.

[B49] Vincent R, Bjarnason SG, Adamson IY, Hedgecock C, Kumarathasan P, Guenette J, Potvin M, Goegan P, Bouthillier L (1997). Acute pulmonary toxicity of urban particulate matter and ozone. Am J Pathol.

[B50] Vincent R, Goegan P, Johnson G, Brook JR, Kumarathasan P, Bouthillier L, Burnett RT (1997). Regulation of promoter-CAT stress genes in HepG2 cells by suspensions of particles from ambient air. Fundam Appl Toxicol.

[B51] Vincent R, Kumarathasan P, Goegan P, Bjarnason SG, Guenette J, Berube D, Adamson IY, Desjardins S, Burnett RT, Miller FJ, Battistini B (2001). Inhalation toxicology of urban ambient particulate matter: acute cardiovascular effects in rats. Res Rep Health Eff Inst.

[B52] Steerenberg PA, Withagen CE, van Dalen WJ, Dormans JA, Cassee FR, Heisterkamp SH, van Loveren H (2004). Adjuvant activity of ambient particulate matter of different sites, sizes, and seasons in a respiratory allergy mouse model. Toxicol Appl Pharmacol.

[B53] Knaapen AM, Albrecht C, Becker A, Hohr D, Winzer A, Haenen GR, Borm PJ, Schins RP (2002). DNA damage in lung epithelial cells isolated from rats exposed to quartz: role of surface reactivity and neutrophilic inflammation. Carcinogenesis.

[B54] Cassee FR, Boere AJF, Fokkens PHB, Leseman DLAC, Sioutas C, Kooter IM, Dormans JAMA Inhalation of concentrated particulate matter produces pulmonary inflammation and systemic biological effects in compromised rats. J Toxicol Environ Health.

